# Retraction: Residues 240–250 in the C-Terminus of the Pirh2 Protein Complement the Function of the RING Domain in Self-Ubiquitination of the Pirh2 Protein

**DOI:** 10.1371/journal.pone.0256909

**Published:** 2021-08-26

**Authors:** The PLOS ONE Editors

Following the publication of this article [1], concerns were raised regarding results presented in Figures 1, 2, 3, and 4. Specifically,

- The second panel of Figure 1A appears similar to the second panel of Figure 1D, despite being used to represent different experimental conditions.
- Lanes 1–4 of the third panel of Figure 1A appear similar to lanes 4–7 of the second panel of Figure 3C, despite being used to represent different experimental conditions.
- The third panel of Figure 1B appears similar to lanes 1–3 of the third panel of Figure 3C, despite being used to represent different experimental conditions.
- The third left panel (E2 anti-His) of Figure 2C appears similar to the third middle panel (Actin) of Figure 4A when rotated 180°.
- The second left panel (E1 anti-His) of Figure 2C appears similar to the third left panel (E2 anti-His) of Figure 2C when flipped vertically, and to the third panel of Figure 4D when flipped horizontally, despite all three panels being used to represent different experimental conditions.
- Lanes 3–5 of the first left panel (Ub-species) of Figure 4A appear similar to the first right panel (Ub-species) of Figure 4A, despite at least one lane being used to represent different experimental conditions.
- Lanes 3–5 of the second left panel (Anti GST) of Figure 4A appear similar to the second right panel (Anti GST) of Figure 4A, despite some lanes being used to represent different experimental conditions. In addition, the second left panel (Anti GST) of Figure 4A appears to show 5 lanes, whereas the labels suggest 6 samples.
- The third panel (Pirh2 anti-GST) appears similar to the fifth panel (E2 anti-His), despite being used to represent different experimental conditions.

The authors stated that several panels were accidentally duplicated during figure preparation, and indicated that the original blots underlying the panels of concern are no longer available due to the time that has elapsed since the experiments were conducted. The authors provided replacement figures but in the absence of the original data underlying the published results, these were insufficient to resolve the concerns raised with the article.

In light of the concerns affecting multiple figure panels that question the integrity of these data, the *PLOS ONE* Editors retract this article.

RPL did not agree with the retraction. RAZ, HW, and CS either did not respond directly or could not be reached.
